# Apoptosis Sensitization by Euphorbia Factor L1 in ABCB1-Mediated Multidrug Resistant K562/ADR Cells

**DOI:** 10.3390/molecules181012793

**Published:** 2013-10-16

**Authors:** Jian-Ye Zhang, Min-Ting Lin, Tao Yi, Yi-Na Tang, Lan-Lan Fan, Xi-Cheng He, Zhong-Zhen Zhao, Hu-Biao Chen

**Affiliations:** 1 School of Chinese Medicine, Hong Kong Baptist University, 7 Baptist University Road, Kowloon Tong, Kowloon, Hong Kong; E-Mails: jianyez@163.com (J.-Y.Z.); yitao@hkbu.edu.hk (T.Y.); 11467312@life.hkbu.edu.hk (Y.-N.T.); fanlanlan1024@gmail.com (L.-L.F.); hexicheng53@163.com (X.-C.H.); zzzhao@hkbu.edu.hk (Z.-Z.Z.); 2 School of Pharmaceutical Sciences, Guangzhou Medical University, 195 Dongfeng Road West, Guangzhou 510000, China; E-Mail: minting113@sina.com

**Keywords:** ABCB1, multidrug resistance, apoptosis, Euphorbia factor L1

## Abstract

In this article, reversal activities of Euphorbia factor L1 (EFL1) against ABCB1-mediated multidrug resistance (MDR) and apoptosis sensitization in K562/ADR cells are reported. EFL1 decreased the IC_50_ values of anticancer agents in K562/ADR cells over-expressing ABCB1. However, EFL1 did not affect the IC_50_ values of anticancer agents in sensitive K562 cells. Additionally, EFL1 increased the intracellular accumulation of rhodamine 123 and doxorubicin in K562/ADR cells without affecting their accumulation in K562 cells. Furthermore, EFL1 sensitized the apoptosis triggered by vincristine in K562/ADR cells via mitochondrial pathway, as confirmed by Annexin V-FITC/PI detection and western blot. At the same time, EFL1 did not influence the apoptosis induced by vincristine in K562 cells. Western blot results showed that EFL1 did not affect the phosphorylation level of AKT and ERK in K562 and K562/ADR cells. Finally, EFL1 did not down-regulate protein expression of ABCB1.

## 1. Introduction

Multi-drug resistance (MDR), which refers to the resistance of cancer cells to multiple structurally and mechanistically unrelated anticancer drugs, is the major obstacle to successful cancer chemotherapy in the clinic [1,2]. P-glycoprotein (P-gp, ABCB1), a member of the ATP-binding cassette (ABC) family, exports structurally diverse compounds from cells through a process driven by ATP hydrolysis [3,4]. High expression and activity of ABCB1 has been linked to the efflux of chemotherapeutic drugs in cancer cells. Indeed, ABCB1 acting as a drug efflux pump is the most important cause of MDR [5,6].

It is well established that many chemotherapeutic agents exert anticancer activity by inducing apoptosis. Most chemotherapeutic agents applied in the treatment of hematologic malignancies cells can induce apoptosis, but MDR tumor cells are generally resistant to apoptosis induction [7,8] and the resistance of leukemic cells to chemotherapy-induced apoptosis remains the most serious problem in the treatment of leukemia [9,10,11].

**Figure 1:**
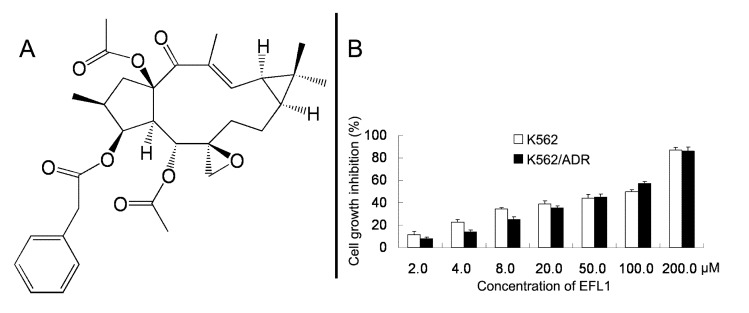
Chemical structure of Euphorbia factor L1 (**A**) and cell growth inhibition results in K562 and K562/ADR cells (**B**).

Currently, the most attractive strategy for overcoming MDR is to use sensitizer or reversal agents, which are combined with chemotherapeutic drugs [12]. Great effort has been taken to find reversal agents from natural products [13,14,15]. In our previous study, we have found that lathyrane diterpene Euphorbia factor L1 (EFL1, Figure 1A) from seeds of *Euphorbia lathyris*, can reverse ABCB1-mediated MDR by inhibiting the efflux function of ABCB1 [16]. In this article, the apoptosis sensitization effect of EFL1 in ABCB1-mediated MDR K562/ADR cells was reported.

## 2. Results and Discussion

Chemotherapy is a valuable tool used in cancer treatment. However, the emergence of cancer cell resistance to chemotherapy often undermines treatment efficacy. Recently, investigators have carried out numerous studies on drug resistance reversal in cancer cells [17]. The major mechanism of resistance is the over-expression of drug efflux pumps, such as P-glycoprotein (ABCB1, P-gp). Multi-drug resistance (MDR) in hematological malignancies is also the main reason of chemotherapy failure [18,19]. One strategy for reversal of MDR in cells over-expressing ABC transporters is the combined use of anticancer drugs with modulators.

We examined the cytotoxicity of EFL1 (Figure 1A) alone in different cell lines by the MTT assay. The IC_50_ values were 33.86 ± 2.51, 39.64 ± 2.93 μM to K562 and K562/ADR cells (treatment time of 96 h), respectively (Figure 1B). More than 90% of cells were viable at the concentrations of EFL1 up to 10.0 μM in all cells under experiments (treatment time of 72 h). We selected EFL1 of 2.5, 5.0 and 10.0 μM to assess reversal of MDR *in vitro* (treatment time of 72 h).

In our study, multidrug-resistant K562/ADR cells were less sensitive to adriamycin cytotoxicity and accumulated less adriamycin than K562 cells (Table 1). The indicated concentrations of EFL1 were chosen for combination treatment with known anticancer drugs acting as substrates of ABCB1, such as Vincristine (VCR) and doxorubicin (DOX). Our data showed that EFL1 dose-dependently enhanced the cytotoxicity of tested anticancer drugs in MDR cells. EFL1 of 2.5, 5.0 and 10.0 μM showed 1.74, 3.79 and 5.88 reversal fold against resistance to DOX in K562/ADR cells, respectively. EFL1 of 2.5, 5.0 and 10.0 μM showed 2.76, 5.06 and 8.47 reversal fold against resistance to VCR in K562/ADR cells, respectively. However, in drug sensitive K562 cells, the cytotoxicity generated by VCR or DOX was unaffected at the presence of EFL1. To evaluate substrate specificity of the transporter, cisplatin, which is not the substrate of ABCB1, was selected as the control [16]. Intriguingly EFL1 did not significantly alter the IC_50_ values of cisplatin in parental sensitive and ABCB1-mediated MDR cells. These results suggested that EFL1 strongly enhanced the sensitivity of ABCB1-overexpressiong MDR cells to conventional chemotherapeutic agents, while EFL1 did not affect the sensitivity of parental sensitive cells.

**Table 1:** Effects of EFL1 on reversing ABCB1-mediated drug resistance.

	IC_50_ ± SD (μM) (fold-reversal)	
	K562	K562/ADR (ABCB1)
VCR	0.032 ± 0.001	3.261 ± 0.412
+ 2.5 μM EFL1	0.032 ± 0.001 (1.00)	1.184 ± 0.027 ** (2.76)
+ 5.0 μM EFL1	0.033 ± 0.001 (0.97)	0.644 ± 0.015 ** (5.06)
+ 10.0 μM EFL1	0.031 ± 0.001 (1.03)	0.385 ± 0.013** (8.47)
Cisplatin	3.642 ± 0.135	3.895 ± 0.167
+ 10.0 μM EFL1	3.510 ± 0.153 (1.04)	3.933 ± 0.204 (0.99)
DOX	0.526 ± 0.0446	14.302 ± 1.237
+ 2.5 μM EFL1	0.513 ± 0.044 (1.02)	8.210 ± 0.068 **(1.74)
+ 5.0 μM EFL1	0.505 ± 0.040 (1.02)	3.773 ± 0.045 **(3.79)
+ 10.0 μM EFL1	0.502 ± 0.040 (1.05)	2.430 ± 0.072** (5.88)

* and ** represent significance at *p* < 0.05 and *p* < 0.01, respectively.

**Figure 2:**
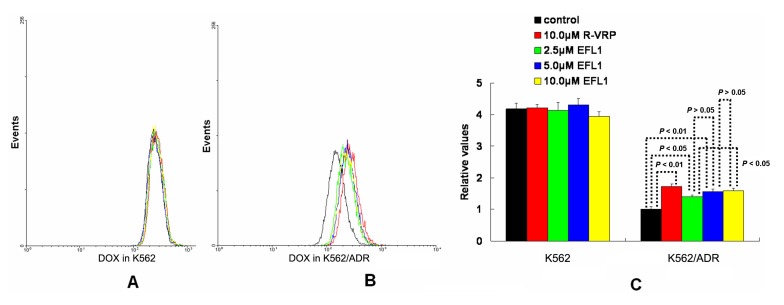
Effects of EFL1 on the accumulation of DOX in K562 and K562/ADR cells. K562 and K562/ADR cells were incubated with 0, 2.5, 5.0 and 10.0 μM EFL1 at 37 °C for 3 h. Then 10 μM DOX of final concentration was added for another 3 h incubation. Intracellular fluorescence was analyzed by flow cytometry with the excitation wave length of 488 nm. R-VRP of 10.0 μM of final concentration was used as the positive control. (**A**) accumulation of DOX in K562 cells. (**B**) accumulation of DOX in K562/ADR cells. (**C**) data analysis of **A** and **B**. All experiments were repeated three times. The relative value was calculated by dividing the fluorescence intensity of sensitive or corresponding drug resistance cells by that of the drug resistance cells without treatment of R-VRP or EFL1, respectively. Columns, means of triplicate determinations. * and ** represent significance at *p* < 0.05 and *p* < 0.01, respectively.

To investigate the related mechanisms, we examined whether EFL1 affected the accumulation of DOX in parental sensitive and ABCB1-mediated MDR cells. The results (Figure 2) showed that EFL1 increased the accumulation of DOX in K562/ADR cells, as indicated by the significantly higher fluorescence of DOX assayed by flow cytometry. Herein, the *R*-enantiomer of verapamil (R-VRP), being an inhibitor of ABCB1 and reversal agent against ABCB1-mediated MDR, was applied as positive control. In K562/ADR cells, the intracellular accumulation of DOX was enhanced to 1.73-, 1.40-, 1.57- and 1.59-fold *vs.* control for 10.0 μM R-VRP, 2.5, 5.0 and 10.0 μM EFL1, respectively. However, EFL1 did not increase the intracellular accumulation of DOX in K562 cells. These results demonstrated that EFL1 was able to interfere with ABCB1-mediated transport.

**Figure 3:**
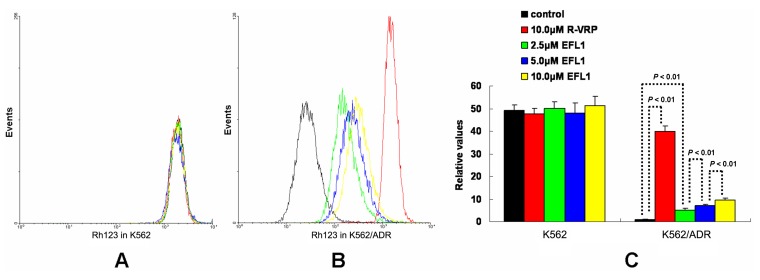
Effects of EFL1 on the accumulation of Rh123 in K562 and K562/ADR cells. Indicated cells were incubated with 0, 2.5, 5.0 and 10.0 μM EFL1 at 37 °C for 3 h. Subsequently, 5 μM Rh123 of final concentration was added for another 0.5 h incubation. Intracellular fluorescence was determined by flow cytometry with the excitation wavelength of 488 nm. R-VRP of 10.0 μM of final concentration was added as the positive control. (**A**) accumulation of Rh123 in K562 cells. (**B**) accumulation of Rh123 in K562/ADR cells. (**C**) data analysis of **A** and **B**. All these experiments were carried out for three times. The relative value was calculated by dividing the fluorescence intensity of sensitive or corresponding drug resistance cells by that of the drug resistance cells without treatment of R-VRP or EFL1, respectively. Columns, means of triplicate determinations. * and ** represent significance at *p* < 0.05 and *p* < 0.01, respectively.

The results of Figure 2 indicate that EFL1 could increase intracellular accumulation of ABCB1 substrates. To confirm this, accumulation of rhodamine 123 (Rh123) was determined. Rh123 is also a substrate of ABCB1. At the same time, Rh123 is a fluorescent dye, which can be detected by flow cytometry. Figure 3 showed that EFL1 could significantly increase accumulation of Rh123 in K562/ADR cells (*p* < 0.01 *vs.* control) and did not affect that in K562 cells (*p* > 0.05 *vs.* control). In K562/ADR cells, the intracellular accumulation of Rh123 was increased to 39.85, 5.06, 7.26 and 9.56 fold *vs.* control for 10.0 μM R-VRP, 2.5, 5.0 and 10.0 μM EFL1, respectively (Figure 3).

**Figure 4:**
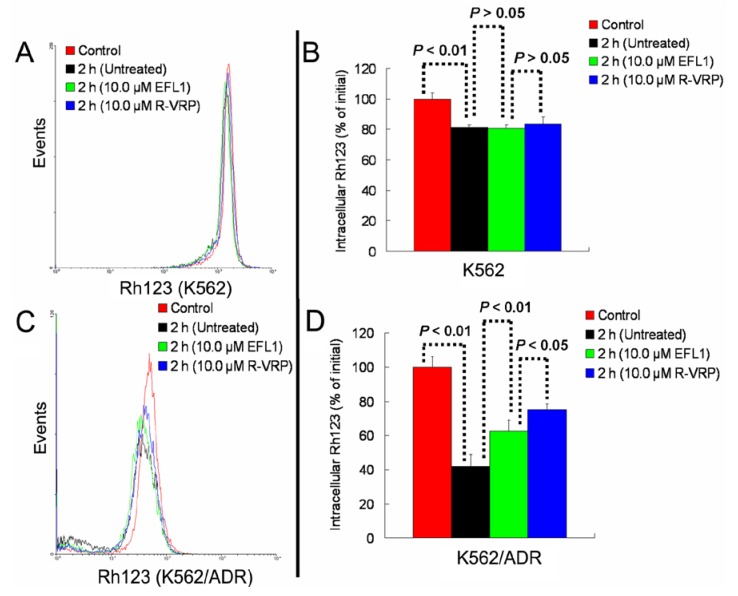
EFL1 inhibited the efflux function of K562/ADR cells and did not influence that of K562 cells. After K562 or K562/ADR cells were treated with 5 μM Rh123 at 37 °C for 30 min, the cells were washed twice by ice-cold PBS and then maintained at 37 °C and absence of Rh123 with culture media containing 10 μM EFL1 or not. At time of 2 h, cells were gathered and washed twice with ice-cold PBS. Subsequently, cells were determined by flow cytometry. (**A**) Rh123 efflux in K562 cells. (**B**) data analysis of **A**. (**C**) Rh123 efflux in K562/ADR cells. (**D**) data analysis of **C**. Columns, means of triplicate determinations. * and ** represent significance at *p* < 0.05 and *p* < 0.01, respectively.

Taken together, Figure 2 and Figure 3 provided support for results of Table 1. Furthermore, our results exhibited that EFL1 inhibited efflux of Rh123 in K562/ADR cells (Figure 4C,D). At the same time, EFL1 did not affect the efflux of Rh123 in K562 cells (Figure 4A,B). In the absence of Rh123, intracellular Rh123 in K562 cells at time of 2 h was 81.16 ± 1.82%, 80.83 ± 2.12% and 83.68 ± 4.47% of control (initial) for group of untreated, 10.0 μM EFL1 and 10.0 μM VRP, respectively. At the absence of Rh123, intracellular Rh123 in K562/ADR cells at time of 2 h was 42.16 ± 6.78%, 62.62 ± 6.34% and 75.33 ± 4.24% of control (initial) for group of untreated, 10.0 μM EFL1 and 10.0 μM VRP, respectively. 

These results indicated that EFL1 could inhibit the efflux function of ABCB1 in K562/ADR cells. It has been proved that EFL1 could increase the ATPase activity of ABCB1 stimulated by VRP by the concentration-dependent manner, acting similarly as verapamil (VRP) and Vandetanib [16]. These indicated that EFL1 reversed ABCB1-mediated MDR via inhibiting function of ABCB1.

**Figure 5:**
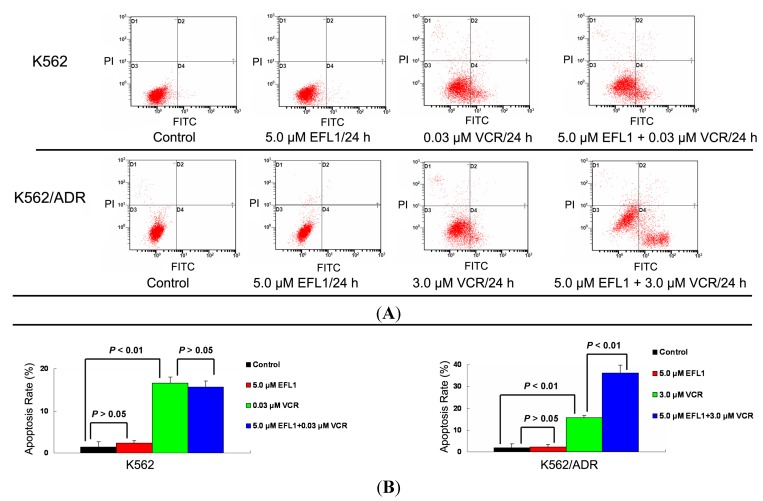
Apoptosis sensitization of EFL1 in K562/ADR cells. After cells were treated with indicated drugs for 24 h, cells were collected washed twice with ice-cold PBS. Then 5 × 10^5^ cells were resuspended with 0.5 mL binding buffer containing Annexin-V and PI for 30 min at 37 °C in the dark. After that, the apoptosis was detected by flow cytometry. (**A**) EFL1 sensitized the apoptosis induced by VCR in K562/ADR cells. However, EFL1 did not alter the apoptosis triggered by VCR in K562 cells. (**B**) data analysis of **A**. Columns, means of triplicate determinations. * and ** represent significance at *p* < 0.05 and *p* < 0.01.

VCR has been applied to treat leukemia and other cancers, where apoptosis induction is involved in the mechanism of action [20]. Herein, apoptosis induced by VCR in K562 and K562/ADR was investigated. The IC_50_ values for K562 and K562/ADR at the absence of reversal agents were applied, respectively. The results of Annexin V-FITC/PI staining and flow cytometry assay showed that EFL1 sensitized the apoptosis induced by VCR in K562/ADR cells (Figure 5). However, EFL1 did not alter the apoptosis triggered by VCR in K562 cells (Figure 5). After K562 cells were treated for 24 h, the apoptosis rate was 1.4 ± 1.2%, 2.3 ± 0.6%, 16.6 ± 1.4% and 15.7 ± 1.4% for control, 5.0 μM EFL1, 0.03 μM VCR and 5.0 μM EFL1+0.03 μM VCR, respectively. After K562/ADR cells were treated for 24 h, the apoptosis rates were 1.9 ± 1.6%, 2.4 ± 1.0%, 15.5 ± 1.2% and 36.2 ± 3.6% for control, 5.0 μM EFL1, 3.0 μM VCR and 5.0 μM EFL1 + 3.0 μM VCR, respectively. These results were consistent with data of Figure 2, Figure 3 and Figure 4 and Table 1. Apoptosis sensitization mediated by other reversal agents has been reported before [7]. Our results suggested that EFL1 showed similar apoptosis sensitization effects.

**Figure 6:**
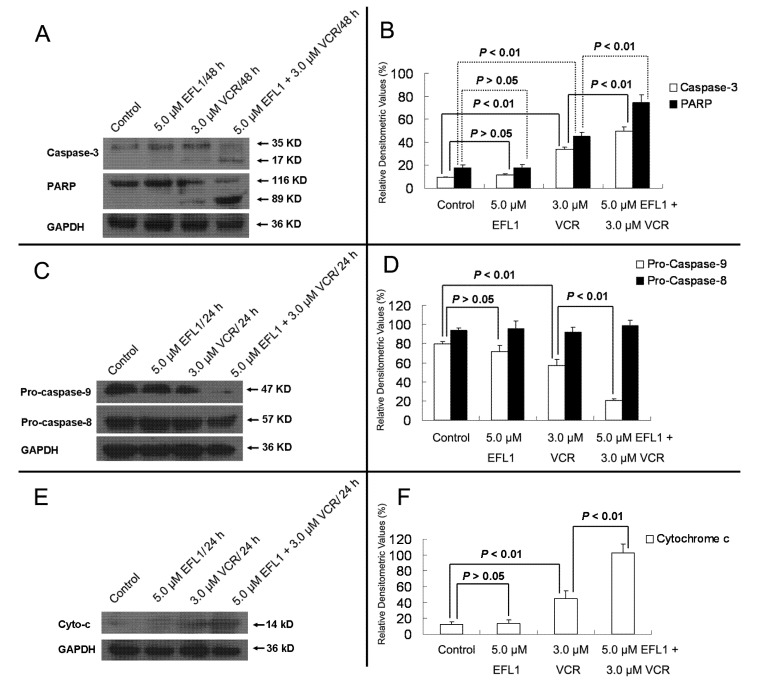
Western blot results of protein related to apoptosis in K562/ADR cells. (**A**) after K562/ADR cells were treated by indicated compounds for 48 h, the total protein was prepared and western blot experiments were carried out. (**B**) Densitometric analysis of western blot results of **A**. (**C**) and (**E**) after K562/ADR cells were treated with indicated compounds for 24 h, whole cell lysates or subcellular fractionation was prepared for western blot. Then, Caspase-8, -9 and cytochrome *c* were determined by western blot. (**D)** and (**F)** Densitometric analysis of western blot results of (**C**) and (**E**), respectively. The values were calculated as (the gray density of investigated protein /the gray density of GAPDH) ×100%. GAPDH detection was applied to confirm the equal protein loading.The results were expressed as mean ± SEM of three experiments. * and ** represent significance at *p* < 0.05 and *p* < 0.01.

Furthermore, western blot results confirmed that EFL1 strengthened the activation and cleavage of Caspase-3 and PARP in K562/ADR (Figure 6). After K562/ADR cells were exposed to tested compounds for 48 h, densitometric ratios (%) of activated Caspase-3/GAPDH were 9.23 ± 0.83%, 11.50 ± 1.11%, 33.54 ± 2.16% and 49.90 ± 3.56% for control, 5.0 μM EFL1, 3.0 μM VCR and 5.0 μM EFL1 + 3.0 μM VCR, respectively. Densitometric ratios (%) of cleaved PARP/GAPDH were 17.61 ± 2.39%, 17.80 ± 3.03%, 44.98 ± 3.57% and 74.82 ± 6.62% for control, 5.0 μM EFL1, 3.0 μM VCR and 5.0 μM EFL1 + 3.0 μM VCR, respectively. The results indicated that EFL1 increased the activation and cleavage of Caspase-3 and PARP caused by VCR in K562/ADR cells (Figure 6A,B). Caspase-3 is the executive Caspase leading to cleavage of PARP and other vital proteins in various cells and the cleavage of PARP was observed in our study (Figure 6). Detection of the decrease of the native 116-kDa enzyme and appearance of the 89-kDa fragment of PARP cleavage can be served as a sensitive indicator that cells are undergoing apoptosis [21].

Herein, the apoptosis pathway was further investigated, including detection of Caspase-9 and -8 (Figure 6C,D). The results indicated that activation of Caspase-9 was involved by showing decrease of Pro-Caspase-9 when K562/ADR cells were treated with VCR or VCR+EFL1 (*p* < 0.01 *vs.* control). On the other hand, decrease of Pro-Caspase-8 was not observed (*p* > 0.05 *vs.* control). It has been reported that activation of Caspase-8 is inhibited by ABCB1 [7], which is consistent with our research. Moreover, release of cytochrome *c* was detected in our research (Figure 6E,F). After K562/ADR cells were treated for 24 h, Densitometric ratios (%) of cytochrome *c*/GAPDH were 11.64 ± 4.08%, 13.69 ± 4.50%, 45.17 ± 9.67% and 102.65 ± 11.56% for control, 5.0 μM EFL1, 3.0 μM VCR and 5.0 μM EFL1 + 3.0 μM VCR, respectively. Our results exhibited that EFL1 could significantly enhance release of cytochrome *c* and then stimulate activation of Caspase-9 (*p* < 0.01).

**Figure 7:**
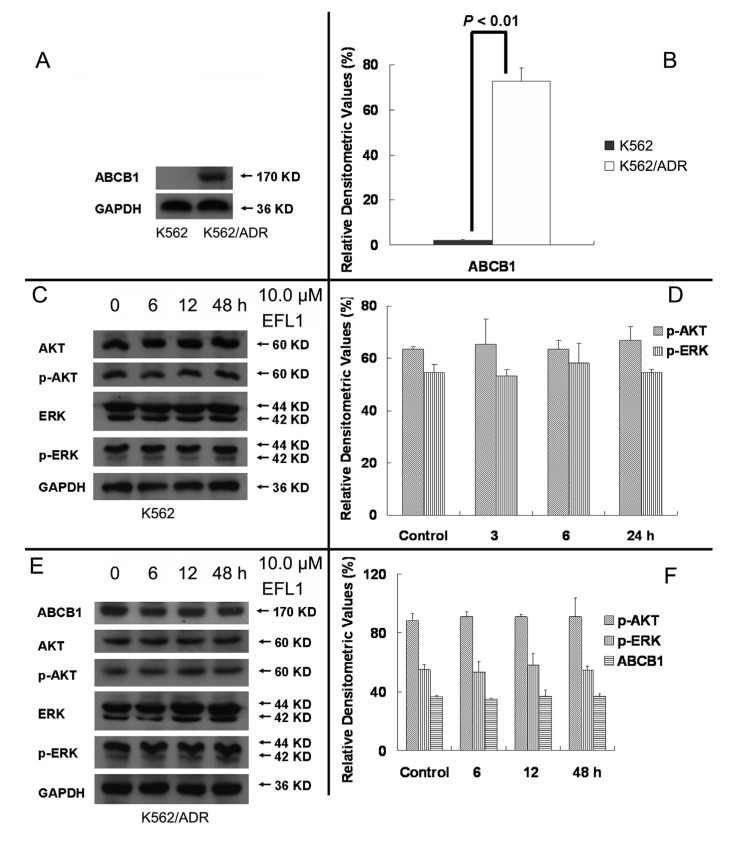
EFL1 did not alter expression of p-AKT, p-ERK and ABCB1. (**A**) protein expression of ABCB1 in K562 and K562/ADR cells. (**B**) Densitometric analysis of western blot results of A. The values were calculated as (the gray density of ABCB1/the gray density of GAPDH) × 100%. (**C**) protein expression of AKT, p-AKT, ERK and p-ERK in K562 cells. (**D**, Densitometric analysis of western blot results of C. The values were calculated as (the gray density of p-AKT or p-ERK/the gray density of AKT or ERK) × 100%. (**E**) protein expression of AKT, p-AKT, ERK and p-ERK in K562/ADR cells. (**F**) Densitometric analysis of western blot results of E. The values were calculated as (the gray density of p-AKT or p-ERK/the gray density of AKT or ERK) × 100% or (the gray density of ABCB1/the gray density of GAPDH) × 100%. The results were expressed as mean ± SEM of three experiments. * and ** represent significance at *p* < 0.05 and *p* < 0.01. GAPDH of MW 36 KD detection was applied to confirm equal protein loading.

More and more studies have showed that inhibition of AKT and ERK1/2 pathways may decrease the resistance in MDR cancer cells [22,23]. To determine whether EFL1 of reversal concentrations attenuate cell survival signaling pathways, the changes of total and phosphorylation forms of AKT and ERK1/2 were investigated in K562 and K562/ADR cells. The results showed that treatment of 10.0 μM EFL1 for 6, 12, 48 h did not alter the phosphorylation forms of AKT and ERK1/2. The densitometric ratios (%) of p-AKT/AKT (Figure 7C,D) in K562 cells for 0, 6, 12 and 48 h/10.0 μM EFL1 were 63.64 ± 0.91%, 65.38 ± 9.85%, 63.41 ± 3.56% and 66.89 ± 5.53%, respectively (*p* > 0.05). The densitometric ratios (%) of p-ERK/ERK (Figure 7C,D) in K562 cells for 0, 6, 12 and 48 h/10.0 μM EFL1 were 54.65 ± 3.00%, 53.38 ± 2.33%, 58.19 ± 7.59% and 54.63 ± 1.09%, respectively (*p* > 0.05). The densitometric ratios (%) of p-AKT/AKT (Figure 7E,F) in K562/ADR cells for 0, 6, 12 and 48 h/10.0 μM EFL1 were 88.47 ± 4.68%, 90.85 ± 3.32%, 90.81 ± 2.02% and 90.90 ± 12.88%, respectively (*p* > 0.05). The densitometric ratios (%) of p-ERK/ERK (Figure 7E,F) in K562/ADR cells for 0, 6, 12 and 48 h/10.0 μM EFL1 were 81.50 ± 3.80%, 80.04 ± 6.93%, 79.95 ± 7.98% and 81.79 ± 2.55%, respectively (*p* > 0.05).

Receptor tyrosine kinases (RTKs) such as vascular endothelial growth factor receptor (VEGFR) and platelet derived growth factor receptor (PDGFR) play a crucial role in modulating cell proliferation, differentiation and survival by activating downstream signaling proteins such as protein kinase B/AKT and extracellular signal-regulated kinase 1/2 (ERK1/2) [24]. Under current understandings, aberrant activation of RTKs is associated with cancer growth, angiogenesis and metastasis [25]. Moreover, several studies have disclosed that activation of AKT and/or ERK pathways is related to resistance to conventional chemotherapeutic drugs [26,27]. Our results demonstrated that total and phosphorylation forms of AKT and ERK remained unchanged in K562 and K562/ADR cells after exposure to 10.0 μM EFL1 for different time (Figure 7). These observations implied that blockade of AKT and ERK activation was not involved in the reversal of ABCB1-mediated MDR in this study of EFL1. Although other compounds from plants of *Euphorbia* have been reported to inhibit phosphorylation form of AKT and ERK [28,29], our results indicated that EFL1 might not be inhibitor of AKT and ERK.

It is notable that EFL1 did not down-regulated expression of ABCB1 in K562/ADR cells (Figure 7E,F). The results showed that the densitometric ratios (%) of ABCB1/GAPDH (Figure 7A,B) in K562 and K562/ADR cells were 3.19 ± 0.87 and 72.71 ± 5.94, respectively (*p* > 0.01). The densitometric ratios (%) of ABCB1/GAPDH (Figure 7E,F) in K562/ADR cells for 0, 6, 12 and 48 h/10.0 μM EFL1 were 83.42 ± 6.97%, 83.21 ± 1.13%, 84.86 ± 4.55% and 86.34 ± 4.70%, respectively (*p* > 0.05). The data suggested that ABCB1 expression in K562/ADR was much higher than that in K562 cells. Treatment of EFL1 under concentration of 10.0 μM for 6, 12 or 48 h did not change the expression of ABCB1 in K562/ADR cells.

In summary, EFL1 elevated sensitivity to chemotherapeutical drugs (e.g., DOX and VCR) in ABCB1-mediated MDR K562/ADR cells and did not affect the sensitivity of sensitive K562 cells. EFL1 might not be inhibitor of AKT and ERK. Although EFL1 did not down-regulate expression of ABCB1, it could inhibit efflux function of ABCB1 and thus enhance the intracellular accumulation of anticancer drugs. Thereafter, EFL1 strengthened the effect of anticancer agents, including sensitizing the apoptosis induced by VCR via mitochondrial pathway. This implied that EFL1 might be used as chemotherapy sensitizer for MDR cancer cells. It is noteworthy that EFL1 can be served as a lead compound, based on which, more powerful reversal agents will be designed.

## 3. Experimental

### 3.1. Reagents

Euphorbia factor L1 (EFL1, Figure 1A) was isolated from Caper Euphorbia seed and identified as having a purity of more than 98%. RPMI 1640 were products of Gibco BRL (Gaithersburg, MD, USA). Antibodies against Caspase-3, -8, -9, cytochrome *c* and PARP were obtained from Signalway Antibody Co., Ltd. (College Park, MD, USA). Antibodies against glyceraldehyde-3-phosphate dehydrogenase (GAPDH), anti-mouse and anti-rabbit IgG-HRP were products of Kangchen Co. (Shanghai, China). 3-(4,5-Dimethylthiazol-yl)-2,5-diphenyllapatinibrazolium bromide (MTT), rhodamine 123 (Rh123), vincristine (VCR), doxorubicin (DOX) and verapamil *R*-enantiomer (R-VRP) were products of Sigma Chemical Co. (St. Louis, MO, USA). Annexin V-FITC/PI Kit was from KeyGEN Biotech (Nanjing, China). Other routine laboratory reagents of analytical or HPLC grade were obtained from commercial sources.

### 3.2. Cell Lines and Culture Conditions

Human leukemia cell line K562 and its adriamycin-selected ABCB1-overexpressing K562/ADR cells were maintained in RPMI 1640 medium containing 10% (v/v) heat-inactivated new-born calf serum, 100 U/mL penicillin and 100 μg/mL streptomycin at 37 °C in a humidified 5% CO_2_ incubator. K562/ADR cells were cultured in the medium containing 1 μg/mL adriamycin for maintaining MDR phenotype, and maintained in drug-free medium for at least seven days before used [18].

### 3.3. Cell Growth Inhibition Assay

The MTT assay as previously described was performed for the analysis of the cell growth inhibition. The treatment time for reversal experiments (Table 1) was 72 h and that for EFL1 IC_50_ values for K562, K562/ADR cells was 96 h (Figure 1B). The concentrations of inhibition growth by 50% (IC_50_) were calculated from survival curves using the Bliss method. The degree of resistance was calculated by dividing the IC_50_ of the MDR cells by that of the parental sensitive cells. The fold-reversal factor of MDR was calculated by dividing the IC_50_ of the chemotherapeutic drugs in the absence of reversal agent by that obtained in the presence of reversal agent [18].

### 3.4. DOX and Rh 123 Accumulations

The effect of EFL1 on the accumulation of DOX and Rh 123 was measured by flow cytometry as previously described. Briefly, 5 × 10^5^ cells of K562 or K562/ADR were incubated in 6-well plates and allowed to attach overnight. The cells were treated with indicated concentrations of EFL1 at 37 °C for 3 h. Then 10 μM DOX or 5 μM Rh 123 of final concentration was added and the cells were further cultured for another 3 h or 0.5 h, respectively. Cells were then collected and washed twice with ice-cold PBS buffer. Finally, cells were resuspended in PBS buffer for flow cytometric analysis (BD FASCanto, New York, NY, USA) and 1 × 10^4^ cells were counted for the fluorescence intensity. R-VRP was used as the control inhibitor in the experiments [30].

### 3.5. Experiments of Rh123 Efflux

Rh123 efflux experiments were performed by modified methods as described before [16]. After K562 and K562/ADR cells were treated with 5 μM Rh123 at 37 °C for 30 min, the cells were washed twice by ice-cold PBS and then maintained at 37 °C and absence of Rh123 with culture media containing 10 μM EFL1 or not. At time of 2 h, cells were gathered and washed twice with ice-cold PBS. Finally, cells were resuspended in ice-cold PBS buffer for flow cytometric analysis (BD FASCanto) immediately and the fluorescence intensity was determined [16].

### 3.6. Annexin V-FITC/PI Assay

Apoptosis rate was determined by measuring surface exposure of phosphatidylserine in apoptotic cells with Annexin V-FITC/PI apoptosis detection kit according to the manufacturer’s instruction. After K562 and K562/ADR cells were seeded in 6-well plate for 24 h, 0 or 5 μM EFL1 was added to the cells. Three hours later, indicated concentrations of VCR were added to the cells and the culture continued for 24 h. Then, the cells were collected and washed twice with ice-cold PBS. 5 × 10^5^ cells were resuspended with 0.5 mL binding buffer containing Annexin-V (1:50 according to the manufacturer’s instruction) and 40 ng/sample of PI for 30 min at 37 °C in the dark. The number of viable, apoptotic and necrotic cells were quantified by flow cytometer (BD FASCanto) and analyzed by the CellQuest software. At least 10,000 cells were analyzed for each sample. The apoptosis rate (%) = (the number of apoptotic cells/the number of total cells observed) × 100% [31,32].

### 3.7. Whole-Cell Lysates and Western Blot Analysis

After 4.0 × 10^6^/well cells were plated on culture dishes (100 × 20 mm) for 24 h, the cells were treated for 48 h. Then, cells were harvested, washed twice with ice-cold PBS. Subsequently, 1.5 mL Eppendorff tubes containing cells was centrifuged at 110 *g* for 5 min and the supernatant was discarded. The pellet was vortexed and 100 µL of 1×loading buffer (50 mM Tris-Cl (pH 6.8), 10% glycerol, 2% sodium dodecylsulphate, 0.25‰ bromphenol blue, 0.1 M dithiothreitol ) for every 5 × 10^6^ cells was added. After being heated at 100 °C for 20 min, the lysates in the Eppendorff were centrifuged at 15, 000 g for 10 min and the supernatant was collected. Equal amounts of lysate protein was separated on 8%–12% sodium dodecylsulfate–polyacrylamide gel electrophoresis (SDS-PAGE) and transferred onto PVDF membrane (Millipore, Billerica, MA, USA). The nonspecific binding sites were blocked with TBST buffer (500 mM NaCl, 20 mM Tris–HCl (pH 7.4), and 0.4% Tween 20) containing 5% nonfat dry milk for 2 h at room temperature. Subsequently, the membranes were incubated overnight at 4 °C with specific primary antibodies diluted in TBST buffer containing 5% nonfat dry milk. Thereafter, the membranes were washed three times with TBST buffer and incubated at room temperature for 1 h with HRP-conjugated secondary antibody. After three washes with TBST buffer, the immunoblots were visualized by Phototope^TM^-HRP Detection Kit (Cell Signaling, Boston, MA, USA) and exposed to Kodak medical X-ray processor (Kodak, Rochester, NY, USA) [33].

### 3.8. Subcellular Fractionation and Western Blot Analysis of Cytosolic Cytochrome c

After 3.5 × 10^6^/well cells were plated on culture dishes (100 × 20 mm) for 24 h, the cells were treated for 24 h. Then, cells were harvested and washed twice with ice-cold PBS, suspended with 5-fold volume of ice-cold cell extract buffer (20 mM Hepes-KOH (pH 7.5), 10 mM KCl, 1.5 mM MgCl_2_, 1 mM EDTA, 1 mM EGTA, 1 mM DTT, 250 mM sucrose, 0.1 mM PMSF and 0.02 mM aprotinin) and incubated for 40 min at 4 °C. Then the cells were centrifuged at 110 *g* for 10 min at 4 °C; the supernatant was subsequently centrifuged at 15,000 *g* for 15 min at 4 °C and the final supernatant was used as cytosolic fraction. Then 5×loading buffer (250 mM Tris-Cl (pH 6.8), 50% glycerol, 10% sodium dodecylsulphate, 1.25‰ bromphenol blue, 0.5 M dithiothreitol) was added to the above obtained supernatant and the mixture was boiled at 100 °C for 15 min. Thus, the protein solution was applied to identification of cytosolic cytochrome *c* by immunoblotting with 10% SDS-PAGE and blotting onto PVDF membrane. The cytochrome *c* protein was detected by using anti-cytochrome *c* antibody in the ratio of 1:1,000 [33].

### 3.9. Statistical Analysis

Results were performed by t-test or one-way ANOVA with SPSS 13.0 software (SPSS Inc., Chicago, IL, USA). Data were presented as means ± SEM of at least triplicate determinations. * and ** represent significance at *p* < 0.05 and *p* < 0.01, respectively. Densitometric analysis of western blot results was carried out by Image J (NIH, Bethesda, MD, USA).

## 4. Conclusions

**Figure 8:**
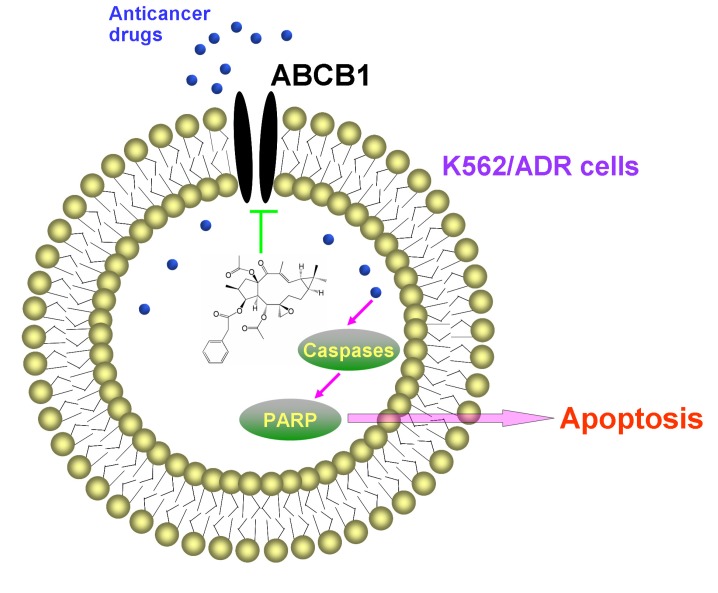
Summary of apoptosis sensitization by EFL1 in K562/ADR cell.

In conclusion, EFL1 could enhance the efficacy of conventional chemotherapeutic agents in MDR K562/ADR cells over-expressing ABCB1 (as summarized in Figure 8). Our results suggest that EFL1 might be useful in combination with ABCB1 substrate chemotherapeutic drugs to overcome multidrug resistance. The mitochondrial pathway involved in the apoptosis sensitization by EFL1. VRP is unavailable in the clinic for its serious cardiovascular side effects when it reaches reversal concentration *in vivo*. We believe that more powerful derivatives active *in vitro* and *in vivo* will be designed on the base of EFL1 as the lead compound.
